# Overview of Facebook Use by Hospitals in Italy: A Nationwide Survey during the COVID-19 Emergency

**DOI:** 10.3390/ijerph18147225

**Published:** 2021-07-06

**Authors:** Beniamino Schiavone, Andrea Vitale, Mena Gallo, Gianlucasalvatore Russo, Domenico Ponticelli, Mario Borrelli

**Affiliations:** 1 General Management Unit, Pineta Grande Hospital, Via Domitiana, km 30/00, 81030 Castel Volturno, CE, Italy; beniamino.schiavone@pinetagrande.it; 2 Research and Development Unit, Pineta Grande Hospital, Via Domitiana, km 30/00, 81030 Castel Volturno, CE, Italy; gallomena1@gmail.com; 3 Communication Office, Pineta Grande Hospital, Via Domitiana, km 30/00, 81030 Castel Volturno, CE, Italy; gianluca.russo@pinetagrande.it; 4 Healthcare Management Unit, Pineta Grande Hospital, Via Domitiana, km 30/00, 81030 Castel Volturno, CE, Italy; domenico.ponticelli@pinetagrande.it (D.P.); mario.borrelli@pinetagrande.it (M.B.)

**Keywords:** health communication, Facebook, social media, COVID-19

## Abstract

*Background*: Facebook is the most popular social network across the world and also allows users access to health information. Our study presents an overview of the official Facebook profiles of hospitals in Italy (*n* = 1351) and how much they are used. *Methods*: All hospitals were surveyed on the number of Facebook posts in May (post-lockdown) and October (second pandemic wave) 2020. The number of followers, the creation date of the official page, and the frequency of publication—that is, the average number of days between two subsequent posts—were determined. *Results*: In Italy, only 28% (*n* = 379) of the hospitals had official Facebook pages, of which 20.6% (*n* = 78) were public hospitals, and 79.4% (*n* = 301) were private hospitals. Of the hospitals with Facebook pages, 49.1% used them every week, and public hospitals published more often. *Conclusions*: Despite the differences between regions and types of management, the number of hospitals in Italy that use Facebook as a tool for the public dissemination of health information is still low. Hospitals should adopt an effective communication strategy using social networks to improve the quality of health care.

## 1. Introduction

In Italy in January 2020, 35 million users (58% of the population) were determined as using social networks, an increase of 6.4% (*n* = 2.1 million) compared to the previous observation period reported in April 2019. Of these users, 29 million (54% of the population) actively used Facebook [1]. Facebook is the most popular social network across the world and allows users to be connected to each other to receive global information on different topics and to generate a comparison between different ideologies. According to the Facebook Report for the second quarter of 2020, there were, on average, 1.79 billion active users daily as of June 30 [2]. The Ottawa Paper, published in 1986 by the WHO, encouraged health institutions to play an active role in health promotion and scientific advances, as well as in the provision of diagnostic–therapeutic activities. The purpose of this universal declaration was to favor the process that allows people to increase their control of and improve their health status [3]. Social networks allow one to reach a wide range of the population, with whom it is also possible to establish a bidirectional interaction, therefore representing a valid tool for health promotion [4,5].

In Italy, in 2008, the first Facebook pages of hospitals began appearing. Today, 28% of hospitals have Facebook pages, indicating a pattern of considerable growth. However, this is still low. This study aims to provide an overview of the existing official Facebook pages of hospitals in Italy by analyzing their activities and engagement with respective followers. The ultimate goal, in addition to demonstrating this phenomenon, is to solicit the hospital communities to equip themselves with this important tool that is frequently requested and used by its users, therefore representing an important contribution to the general public community. It is particularly suitable for promoting a healthy lifestyle and for providing information on health services in order to bring users closer to the world of science and medicine [6]. The new coronavirus SARS-COV2 (COVID-19) pandemic has resulted in the spread of incorrect and uncontrolled information which has severe repercussions on both national and regional health policies, as well as in the daily life of all of us [7].

Especially during this period, the scientific community has gained awareness of the importance of appropriate and valid online communication. It increases the spread of specific messages relating to health protection and promotion, but above all, allows users to strengthen their trust and alliance with health professionals, institutions, and associations of patients [8,9]. Hospitals are certainly authoritative sources of health information, especially because they can communicate through the field the experience of their doctors and nurses [10], as apparent during the lockdown period (9 March to 18 May 2020) and the beginning of the second epidemiological wave, in which in Italy generated an extraordinary demand for health information through the use of web search engines and social media. Hospital Facebook pages could also contribute to the dissemination of communication campaigns promoted by the Ministry of Health, and by monitoring interactions with the public, it will be possible to expand the knowledge of the health needs of the population and to subsequently adapt the healthcare that is on offer [11].

Studying users through social networks, particularly through the interactions between the public and hospitals, is a further opportunity to gather information useful to government institutions, especially for planning strategies in favor of health determinants, while considering the limits derived from the freedom of users to maintain anonymity [12]. There is still a large degree of skepticism regarding the use of social networks by many researchers, who find it difficult to make the most of the potential of this medium in health research [13]; however, the bidirectional exchange of information and the resulting data are enormous. Therefore, an effort is needed to overcome the obstacles that hinder its advance in the scientific community [14].

In this work we aim to answer the following questions:

1. Given the impact of social media on patients’ clinical choices, how much attention is paid by hospitals on the information and scientific communication to patients through social networks?
2. Is the information provided frequently updated?
3. Are there differences in the use of social networks between public and private hospitals in Italy?
4. Are there any virtuous examples of healthcare organizations that are able to communicate effectively on social media?

## 2. Materials and Methods

### 2.1. Data Collection

In this study, all hospitals in Italy were examined (*n* = 1351) according to the updated list of the Ministry of Health as of 30 December 2016, meaning that 32 structures were not analyzed because they had been converted into other health destinations or no longer existed [15]. A Microsoft Excel dataset was constructed using the data extracted for all the official hospital Facebook pages (Supplementary Materials). The extraction of data was performed manually by two people (the first and fourth authors), and no computer tools have been used. This data was cross-sectional in nature. Considering the possibility of a dependent operator error, the acquired data were subsequently revised through a comparison of the data reported in the dataset with the screenshots taken at the time of collection. In creating the dataset, the hospitals were divided into two groups: Public hospitals (*n* = 709) and private hospitals (*n* = 643). In Italy, healthcare is public and refers to the inspiring principles of the Beveridge model, provided by public and private players—the latter represented by corporations. The public group is made up of four subcategories: Hospitals, A.O.U. (university hospitals), I.R.C.C.S. (scientific hospitalization and treatment institutes), and hospital presidiums. In the private group, on the contrary, there are six subcategories: accredited nursing homes, authorized nursing homes, I.R.C.C.S., classified hospitals (generally religious hospitals), university hospitals, and hospitals.

### 2.2. Characteristics and Sampling of the Facebook Pages

To obtain the information necessary to build the dataset, a Facebook account was created, through which the official pages of, or those managed by the hospitals, excluding the unofficial pages created automatically by the social network after the publication of posts by users who have registered using the geographic location of the hospital, were searched for. The pages of individual operating units belonging to a hospital organization were also excluded from the sampling. The data obtained from the observation of all the pages found on the social network were gradually archived in a spreadsheet (Microsoft Excel). Once the data collection had been completed, the analytical phase was carried out based on the parameters collected, namely: (1) number of likes, (2) number of followers, (3) number of posts in May and October, (4) date of creation of the page, and (5) activity of the page. Page activity is described as the average, in days, of time intervals between the most recent and the penultimate posts, and that which is between the penultimate and the third-to-last post (time interval between the survey date and the most recent published post and the time interval between the first and third most recent posts). The number of likes differs slightly from the number of followers of the hospital pages, given that there is a difference in meaning, where the first indicates the satisfaction with the page of users and is a subset of the second value, which, instead, indicates the total number of users who follow the social page.

The frequency of posting on official Facebook pages of hospitals represents how often the hospital publicly post, measured considering the average between the days of the last and penultimate post published, and those of the difference between the penultimate and the third-to-last post published.

Each of the parameters analyzed in the study is represented with graphs and tables that facilitate the understanding of the data, particularly highlighting the use of social networks by hospitals through a comparison between the public sector and the private sector.

### 2.3. Statistical Analysis

An exploratory analysis was carried out using statistical software, R (Developer R core Team, http://www.r-project.org (accessed on 25 May 2021). version 4.1.0), using a choropleth map and bar charts to investigate the main characteristics of hospitals that have an official Facebook page. The analysis continued with contingency tables and chi-square tests to analyze the association between the qualitative variables, such as the type of facility and the type of hospital management with respect to the presence of an official Facebook page. Three-dimensional bar graphs and scatterplots are also shown to visualize the activity of the hospitals during the months of May and October 2020. The analysis ended with a Kruskal–Wallis test to detect differences in the number of followers by type of management and geographic area.

## 3. Results

### 3.1. Distribution of Official Facebook Pages across Italian Hospitals

**Figure 1:**
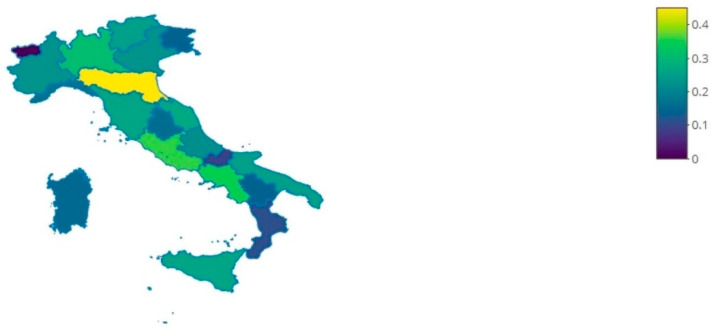
Choropleth map in which the color intensity of the region is relative to the percentage of hospitals that have a Facebook profile.

Figure 1 shows the percentage of hospitals that have a Facebook profile compared to the total number of hospitals for each region, and it is clear that there are some regions far behind, such as Valle d’Aosta, Molise, and Calabria (0–0.11%), while regions such as Emilia-Romagna, Lazio, and Campania have the highest rates (0.45–0.35%).

**Figure 2:**
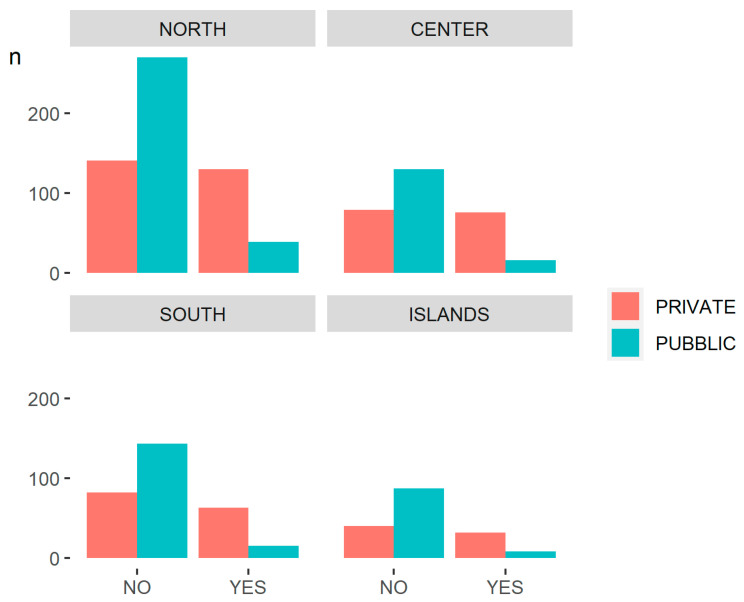
Bar diagram differentiated by geographical area.

In all geographic areas, the number of private hospitals that have an official page is approximately equal to those that do not have a Facebook profile; on the contrary, there is a big difference between public hospitals that have a Facebook page compared to those that do not, and this difference is very evident in hospitals in the north of Italy (Figure 2).

Considering the total list of hospitals in Italy (*n* = 1351), 708 in the public sector, and 643 in the private sector, it was found that 28% (*n* = 379) have an official Facebook page. The distribution of the official hospital pages is as follows: 20.6% (*n* = 78) public hospitals and 79.4% (*n* = 301) private hospitals (Table 1).

The type of hospital with the highest percentage of official pages was shown to be accredited to nursing homes. In addition, there was a significant association with regards to the type of facility and whether it has an official page (Table 2).

**Figure 3:**
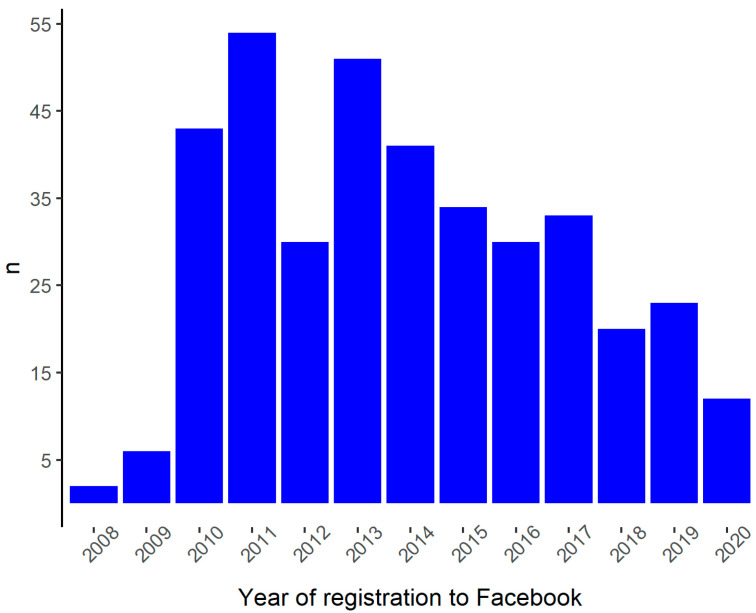
The year is shown on the x-axis, while the number of structures is shown on the y-axis.

In Italy, Facebook arrived in 2008 and has seen an increasing number of registrations since then. After a major increase in the number of Facebook registrations by Italian hospitals recorded in 2010, 2011 reported the highest number. On the contrary, the greatest decrease in registrations occurred in 2012, and in recent years, there have been no significant increases (Figure 3).

### 3.2. Distribution of Posts in May and October

**Figure 4:**
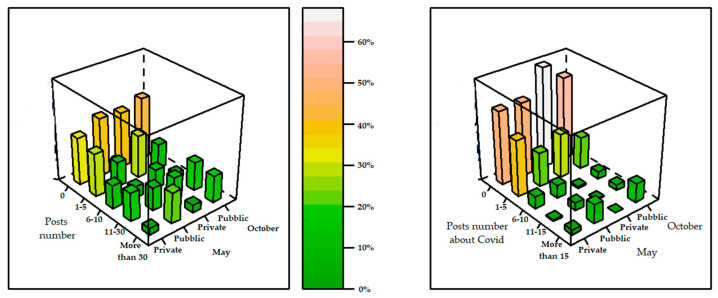
The intensity of the color of the vertical bars refers to the frequency (%) of the categories and increases in a mirror image of the colored bar in the center.

In this section, we analyzed the variables related to the number of posts published in May and October 2020, factorized using the following categories: 0 posts, 1–5 posts, 6–10 posts, 11–30 posts, and more than 30 posts. In the same way, the variables relating to the number of posts referring to COVID-19 were analyzed using the categories: 0 posts, 1–5 posts, 6–10 posts, 11–15 posts, and more than 15 posts. What emerges is that there has not been a change in the frequency of publications, and many of the posts do not concern COVID-19. A very important fact that Figure 4 demonstrates is that, although more private structures have official profiles, it is the public sector that shows a greater commitment to publication, reporting higher frequencies in both May and October for the “more than 30 posts” category, as well as more news on COVID-19.

**Figure 5:**
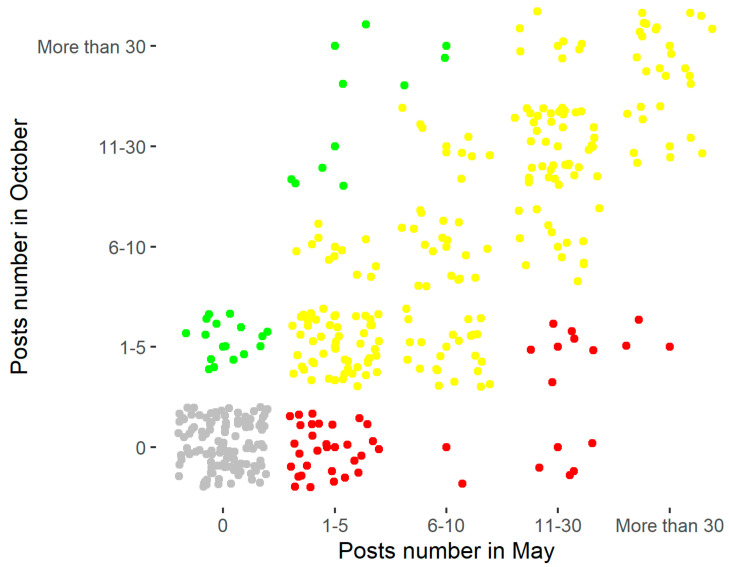
Scatterplot.

As aforementioned, there was no substantial difference in the frequency of publications between May and October 2020. This is also confirmed by Figure 5, in which the gray dots represent the hospitals that did not publish any posts in either May or in October (30%); meanwhile, the yellow dots (53%) indicate the hospitals that did not change their publication frequency during the two months, the red dots show the hospitals that published less in October than in May (11%), and the green dots highlight the hospitals that posted more frequently in October than in May (6%).

### 3.3. Distribution of Publication Frequency

The variable relating to the publication frequency is the average between the difference in days between the last and penultimate posts, and that which was between the penultimate and third-to-last posts. Table 3 shows that public facilities use social media every day or on a weekly basis, while Table 4 statistically describes the frequency of posts.

### 3.4. “Followers” Distribution

Finally, this section reports the descriptive statistics of the continuous variable “number of followers”. Table 5 statistically describes the distribution of followers.

**Figure 6:**
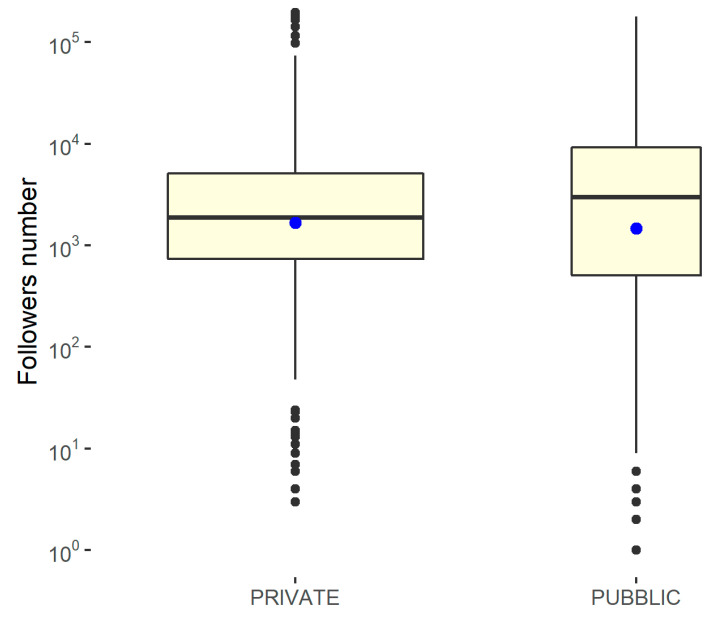
Kruskal–Wallis rank-sum test to determine the differences in the number of followers for the public/private hospitals. Kruskal–Wallis chi-squared = 1.1029, df = 1, *p*-value = 0.2936 (no significant difference).

**Figure 7:**
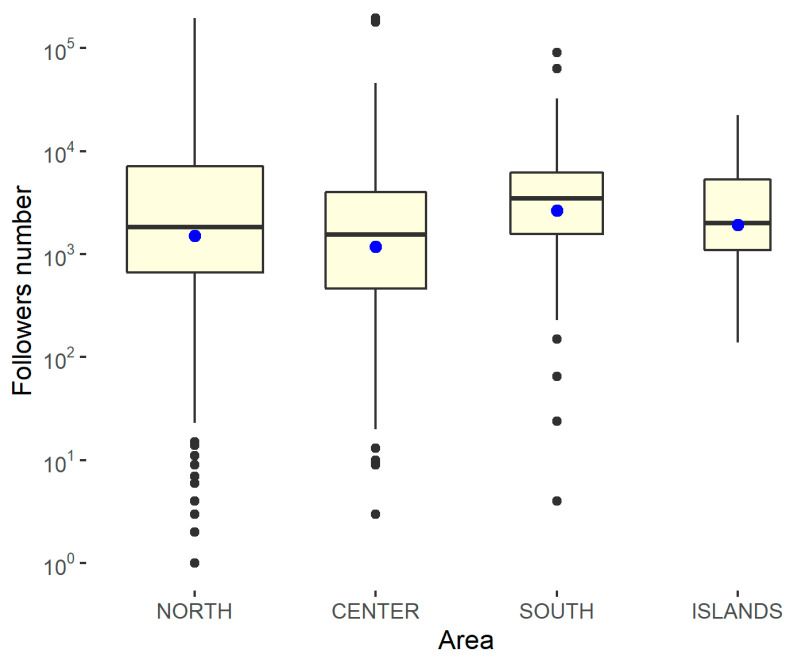
Kruskal–Wallis rank-sum test in the number of followers for the area. Kruskal–Wallis chi-squared = 8.5252, df = 3, *p*-value = 0.03632 (there is a significant difference).

To capture any differences, box graphs are shown, differentiated according to whether the structure is public or private and by geographical area. Since the results of the Shapiro–Wilk normality test, performed using the statistical software R on the variable relating to the number of followers with respect to the geographical area and the type of management, shows a *p*-value of <0.001, the null hypothesis must be rejected, according to which the distribution is normal. Furthermore, the Levene test showed a *p*-value of >0.05, demonstrating that homoskedasticity can be assumed with respect to the geographical area and type of management. Therefore, in order to measure the significance of the differences, it was necessary to resort to non-parametric approaches on the median. In this case, a *p*-value of <0.05 is needed to reach significance. To evaluate the difference in the number of followers, box plots differentiated by the type of feed and geographical area were constructed. Furthermore, a non-parametric approach (Kruskal–Wallis ranksum test) was used to study the statistical significance of the differences, as reported in Figure 6 and Figure 7.

The *p*-value indicates that there was a significant difference with respect to the geographical area; however, it was necessary to analyze all of the possible pairs and to consider an adjusted p-value, as reported in Table 6, from which it can be deduced that there was a significant difference in only the central–south region.

**Figure 8:**
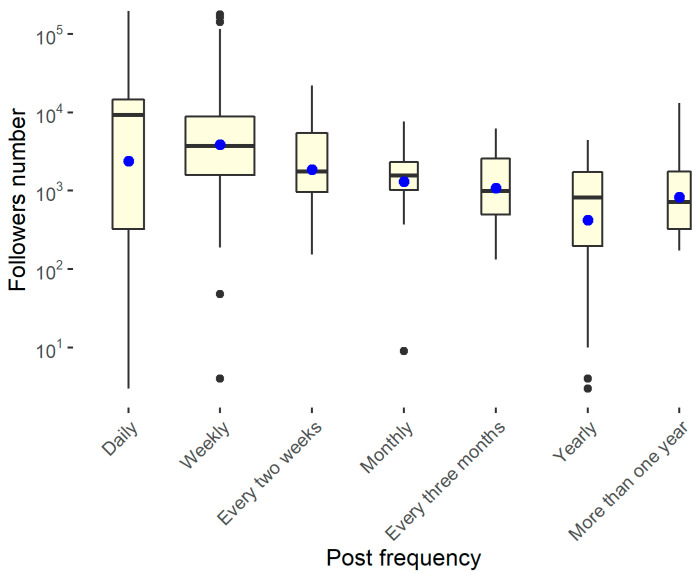
Comparison between the number of followers and post frequency.

Finally, Figure 8 shows that the structures that update the social pages every day or every week are also the profiles that have the most followers.

### 3.5. “Like” Distribution

This section shows the descriptive statistics of the continuous variable “number of likes,” as reported in Table 7.

**Figure 9:**
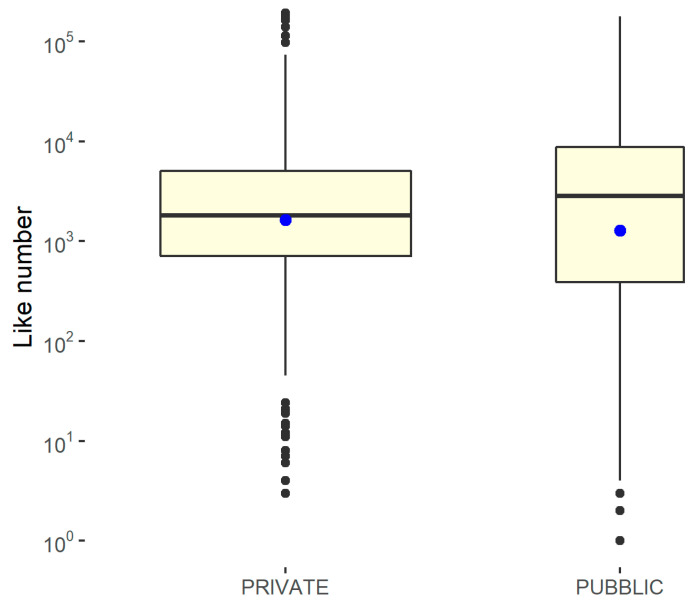
Kruskal–Wallis rank-sum test to determine the differences in the number of likes for the public/private hospitals. Kruskal–Wallis chi-squared = 0.679, df = 1, and *p*-value = 0.41 (no significant difference).

**Figure 10:**
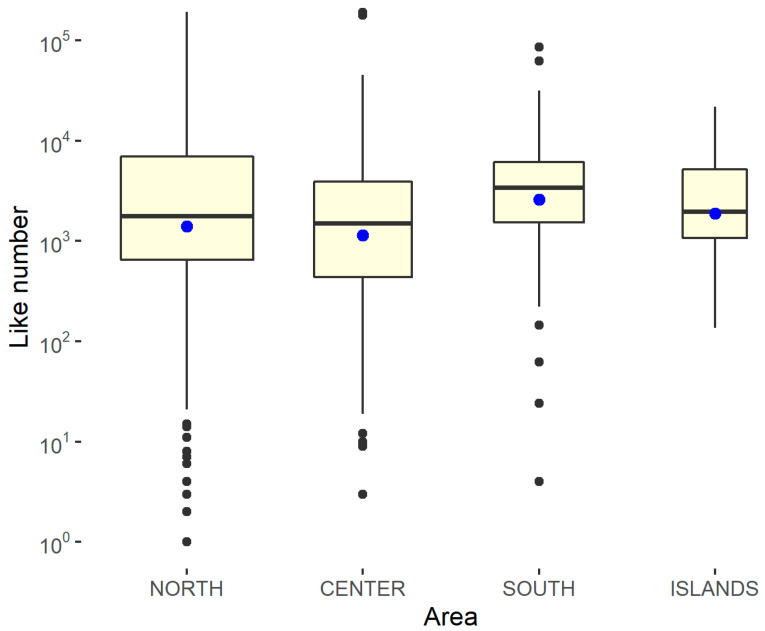
Kruskal–Wallis rank-sum test on the number of likes per area. Kruskal–Wallis chi-squared = 8.8093, df = 3, *p*-value = 0.031 (significant difference).

Since the results of the Shapiro–Wilk normality test performed on this relative variable with respect to the geographical area and type of management showed a *p*-value of <0.001, the null hypothesis that the distribution is normal must be rejected. Furthermore, the Levene test showed a *p*-value of >0.05, demonstrating that homoskedasticity can be assumed with respect to the geographical area and type of management, as reported in Figure 9 and Figure 10 and in Table 8.

**Figure 11:**
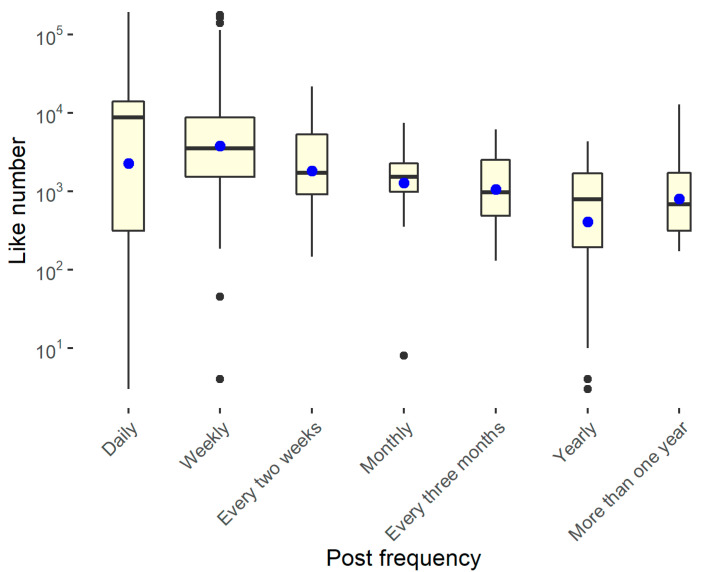
Comparation between like number and frequency of posts.

Figure 11 shows that, as was easily understood, the structures that update the social pages every day or every week are also the profiles that have the most likes.

## 4. Discussion

Social networks are playing a leading role as tools for health promotion, highlighting the importance of adopting a healthy lifestyle [16], as well as in promoting a good relationship [17] between the doctor and the increasingly informed patient.

The main objective of this study is to stimulate healthcare service providers, such as hospitals, to be more active in the proper use of social media. In fact, clinicians should increase the frequency of publication on their Facebook pages on a daily basis and treat with particular emphasis the topics related to risk factors, prevention [16,18], treatment, early diagnosis, and cure, as well as investigate which aspects of these messages generate greater involvement in the public [19]. In the face, more and more people are being encouraged to consider various treatment options and talk to their healthcare professionals [20,21].

In a systematic literature review of empirical studies, Smailhodzic et al. reported that social media use has an even greater impact on doctor-patient relationships and identifies several categories (emotional, information, esteem, network support, social comparison, and emotional expression) of effects in which the 1700 articles analyzed are grouped [17].

In other studies, Rolls et al. reported that social media can also improve patients’ access to health care information and other educational resources [22]. While Dizon et al. observed that doctors promote patient health care education by using social media [23].

Notable, the active role in the communities of social networks [24]. In fact, the hospital clinicians will be the main providers of correct, precise, and validated scientific data. Patients, also aware of the close collaboration between patient associations and hospital clinicians, will be reassured and will not be easy prey to fake news or information of poor quality and reliability [14], often contradictory and lacking in scientific evidence and they can be exposed to various risks [21]. In fact, hospitals represent a source of reliable information, validated and shared by the scientific community, and based on Evidence-Based Medicine (EBM) [25,26]. Therefore, by optimizing the Health Messages on Facebook pages about diseases, treatments, and doctors, many users will be able to find the correct information before planning a visit [27], as well as being able to more easily follow the doctor’s recommendations and stick to the proposed treatment plan, especially if patients become part of a social media support group [28,29]. Several research studies related to health have evaluated the effectiveness of this communication strategy by using social networks [30,31]. There are good examples of social care providers. A noteworthy case is that of Mayo Clinic, engaged in the management of dozens of hospitals in the US and which through various social accounts on various platforms is constantly engaged in disease awareness and prevention. Also, on an international level, the National Cancer Institute [32] and the WHO [33] are also excellent examples of how you can communicate with social networks. Among the Italian institutions, AIFA is actively engaged in the use of social networks for communication to the public [34]. Finally, among the Italian hospitals, it has managed to build a strong presence on social media and in the Emilia Romagna region. The University hospital—Policlinico S.Orsola-Malpighi has distinguished itself as a virtuous example for its health care social media marketing strategy, achieved in just 4 years (from 2016 to 2020) from the opening of its Facebook page, to involve over 105,000 followers, organically, through an emotional communication based on storytelling, which has given life to a loyal community, promoting the “connection” as a “point of relationship“ between clinicians and patients [35].

### 4.1. Principal Findings

This is an overview of the official Facebook pages of the hospitals in Italy as of May and October 2020. According to the results based on the total number of hospitals in Italy (*n* = 1352; 709 in the public sector and 643 of the private sector), only 28% (*n* = 379) had official Facebook fan pages. Of these, 20.6% were public hospitals (*n* = 78) and 79.4% were private hospitals (*n* = 301). Of those hospitals with a Facebook page, 59% (*n* = 224) of them were accredited to nursing homes. Nearly half of the hospitals with a Facebook page (49.1%, *n* = 171) use social media every week, with public hospitals posting the most. Emilia-Romagna was the region identified as having the highest percentage of hospitals with an official Facebook page. However, there were differences relating to the frequency of publication among the hospitals. These differences were evident not so much for the type of structure or the geographical area, but for the type of management (public or private). In fact, more public (34.5%) than private hospitals (5.2%) tended to publish several posts on their official Facebook page, typically daily or weekly. The frequency of the publication of posts relating to COVID-19 was also higher in the public than in the private sector, dedicating more than 50% of the posts to information relating to COVID-19. However, despite the COVID-19 pandemic, there were no substantial differences in terms of creating new Facebook pages for those hospitals that did not have them or between the increase in the number of posts published in the periods of May and October 2020. The non-parametric statistical analysis of the number of followers did not show any significant differences between the types of structure or types of management. There was a significant difference only in the central–south comparison (*p* < 0.01) in terms of followers. More followers visited the pages of those structures that update their Facebook pages more frequently through the publication of informative posts, and these structures receive more “likes” than those of other structures that update their pages less. All of the Facebook fan pages are freely available via the Internet with a Facebook account.

### 4.2. Limitations

Our study shows some limitations. First, social media is dynamic, as the number of “likes” registered, the number of followers, the interactions between users, the views, and the number of posts published in the last month, and social media accounts are subject to continuous change over time. Collecting data over a longer period could allow us to perform a more in-depth analysis and examine trends in Facebook-based hospital-generated content. In fact, another limitation of our research is that we did not assess the issues addressed in the posts published by the hospitals, the differences between hospitals in terms of the types of content they publish on their Facebook pages, and the relationship between the topic covered in the publication and the patient satisfaction rate in response to the different information provided on the hospital pages. Understanding user involvement is important because it can transform one-way information dissemination activities into two-way communication processes. In this way, platforms such as Facebook could add value to the activities of health organizations and to the dissemination of information essential for promoting the well-being of the community. Second, we observed that several hospitals did not present an official Facebook page, but departments within those hospitals had created their own fan page as a tool to disseminate information related to health and progress in specific fields. These specialized pages, with their content, frequently being updated, their involving users in research activities, and their providing information regarding the services offered and the goals achieved, have increased the number of “likes” and shares among users. However, these pages were not considered in this work due to their representation of the entire hospital, which resulted in the loss of important data. Third, we were unable to examine the factors that could affect the content generation process in terms of who is responsible for publishing content on Facebook pages, managing resources, and overseeing the administration duties. In particular, it would be interesting to observe how the content provider affects the type of content shared, the way it is shared, and the resulting level of user engagement. Ultimately, this can affect the overall effectiveness of healthcare organizations’ activities on Facebook and other social media platforms. Fourth, in our research, it was not possible to examine users’ Facebook profiles by collecting information on their gender, race, age, interests, content, and favorite topics, as they were not publicly available. Furthermore, it was not possible to evaluate some statistical data, as they were not publicly available, such as the number of accounts reached, the number of views, user interactions with content, the weekly increase in followers, and, above all, the relationship between the frequency of publications and the percentage of users interested in the information provided on the hospital Facebook pages. Without these data, we cannot know if the number of “likes” was related to the quality of the information produced and/or shared by the hospital, and the users’ satisfaction rate. The frequency with which a page publishes content, allows them to reach an increasingly vast and heterogeneous audience, influencing the levels of engagement of users on social networks. Furthermore, the number of “likes” a page receives is not necessarily related to the level of use of the information provided by hospitals, and patients do not often choose a hospital based on the information on Facebook pages. In fact, patients tend to choose hospitals for their care based on the previous experiences of family or friends. Furthermore, the period in which a page was created could affect the number of followers and total “likes,” as older pages would have had more time to increase their numbers. Therefore, further studies should be conducted and other possible benefits of social media for healthcare professionals and users should be evaluated.

## 5. Conclusions

Several studies have evaluated the Facebook posts and Likes as qualitative indicators of the hospital and of the information published [19,36], and in other cases, the interactions in health research [37].

In other studies, the number of people who have been “convinced” by each information and communication campaign has been quantified, especially on the modification of lifestyles and on all chronic diseases [38], including oncological ones [19,39], where the use of tools as an online community it is very large. The same applies to the need for the strong psychological support that these pathologies require [29,40,41].

This analytical observational study highlighted useful elements for describing the phenomenon, in the panorama of Italian hospitals, of the creation and use of an official Facebook page. Facebook is confirmed as an authoritative communication tool, potentially useful for disseminating information regarding public health.

This study aimed to present an overview of the official Facebook pages of Italian hospitals that use Facebook as a communication tool. Despite the popularity of Facebook, Italian hospitals are still lagging behind in their use of Facebook, not only as a simple and useful tool for promoting public health but also as a tool with which to counter or limit fake news in medicine [42]. In fact, during the COVID-19 health emergency, much fake news and real disinformation campaigns on coronavirus were spread, highlighting the primary role of hospitals in publishing accurate, useful, and scientifically accredited information. Consider that, between April and June 2020, 7 million posts spreading disinformation on coronavirus were removed, while another 98 million posts were labeled as unreliable on the same topic, even if it was not possible to remove them because the violations of the guidelines were not such as to justify this measure [43].

It would be useful to carry out further analyses and to collect different types of data, such as turnover of the structure and access to research funds or demographic characteristics of followers to determine the specific advantages of having a Facebook profile, but it is clear that it is important for a structure to invest in the formation of a team of social media experts to promote and increase the prestige of the structure itself. Many hospitals, still, do not seem to be making good use of social media [44]. The associations of health professionals, in concert with public health bodies, should coordinate so that guidelines [30,31] and recommendations are adopted for the correct use of social media in the health sector. It is fundamental to choose an effective communication strategy [45].

The descriptive analysis conducted in this value highlights that there are more public hospitals that have an official Facebook page, but in both May and October there was a greater commitment of public hospitals to publish more posts and update their official Facebook page accordingly. Likewise, public hospitals have always published more posts related to the COVID-19. Finally, it should be noted that there are no significant differences between public and private management structures in terms of the number of “likes” and followers. These results suggest that it would be advisable, especially for private hospitals, to have a communications office coordinated by a Social Media Manager, whose dedicated multidisciplinary staff are able to frequently disseminate useful, updated, and scientifically accredited information in the health sector. This approach would make it possible to reduce disinformation, especially in times of pandemics, such as the COVID-19 emergency, and to generate greater trust in patients. The reasons for the use and non-use of Facebook by both hospitals and patients require further investigation. In order to achieve and maintain a high-quality level of health, it is necessary to aim for the correct provision of health information, the implementation of public health interventions, and greater patient engagement by the involvement of the community and the activity of the page with numerous initiatives and events for the general public [46]. Additionally, the hospitals should also dedicate sections to research and development activities on their social profiles, including the sharing of scientific publications, in order to allow patients to get informed on the innovative approaches they could have access to at that hospital [47]. Finally, a constant updating of social networks would lead to greater user involvement by reducing disinformation that often places the health and scientific environment in a bad light [48,49]. In fact, considering, in recent years, the average age of those who use Facebook, the use of social networks for precise and timely health information could bring the older generations of the population closer and closer [50].

It is necessary to highlight the social importance for hospitals and, in particular, health professionals, who can act as spokespersons [51]. Additionally, more information and greater hospital transparency—especially of pharmaceutical companies on data, progress, and other things, could greatly reduce the flood of hoaxes that generate panic among people.

## Figures and Tables

**Table 1:** Contingency table and chi-square test.

	Official Page		Total
	NO	YES	
Private	342 53.2%	301 46.8%	643 100%
Public	630 89%	78 11%	708 100%
Total	972 71.9%	379 28.1%	1351 100%

χ^2^ = 212.142, df = 1, φ = 0.398, *p* < 0.001.

**Table 2:** Association between the type of hospital and whether they have an official page.

Type of Structure	Official Page		Total
	NO	YES	
Hospital	98 77.2%	29 22.8%	127 100%
University Hospital A.O.U	16 61.5%	10 38.5%	26 100%
University Hospital Integrated with the NHS	9 75%	3 25%	12 100%
Accredited Nursing Home	264 54.1%	224 45.9%	488 100%
Non-accredited nursing home	27 44.3%	34 55.7%	61 100%
Research Institution	2 66.7%	1 33.3%	3 100%
IRCCS Foundation	5 50 %	5 50%	10 100%
IRCCS	27 48.2%	29 51.8%	56 100%
Qualified institutes for the L.H.U.	7 38.9%	11 61.1%	18 100%
Directly managed hospital	498 95.8%	22 4.2%	520 100%
Classified Hospital	18 64.3 %	10 35.7%	28 100%
University Hospital	1 50%	1 50%	2 100%
Total	972 71.9%	379 28.1%	1351 100%

χ^2^ = 278.656, df = 11, Cramer’s V = 0.454, Fisher’s *p* < 0.001.

**Table 3:** Use of social media every day or on a weekly basis by public facilities.

	Post Frequency							*Total*
	Daily	Weekly	Every Two Weeks	Monthly	Even Three Months	Yearly	More than One Year	
Private	15 5.2%	151 52.1%	33 11.4%	25 8.6%	24 8.3%	26 9%	16 5.5%	290 100%
Public	20 34.5%	20 34.5%	1 1.7%	4 6.9%	5 8.6%	5 8.6%	3 5.2%	58 100%
Total	35 10.1%	171 49.1%	34 9.8%	29 8.3%	29 8.3%	31 8.9%	19 5.5%	348 100%

χ^2^ = 49.843, df = 6, Cramer’s V = 0.376, Fisher’s *p* < 0.001.

**Table 4:** Descriptive statistics of the frequency of posts (SD, Standard Deviation; SE, Standard Error).

*n*	% Missing	Mean	SD	SE	Median	Range
348	8.18	65.50	189.84	10.18	4.50	(0–1649.5)

**Table 5:** Descriptive statistics of the distribution of followers.

Variable	*n*	% Missing	Mean	Sd	SE	Median	Range
Followers number	378	0.26	8602.91	24,749.24	1272.96	1973.00	(1–195,602)

**Table 6:** Pairwise Wilcoxon rank-sum tests in the number of followers for the area.

Group 1	Group 2	n1	n2	Median 1	Median 2	*p*-Value	*p*-Value Adjusted	Significance
NORTH	CENTER	169	92	1830.5	1546.5	0.293	0.879	ns
NORTH	SOUTH	169	78	1830.5	3482.5	0.045	0.224	ns
NORTH	ISLANDS	169	40	1830.5	2006.5	0.782	0.879	ns
CENTER	SOUTH	92	78	1546.5	3482.5	0.003	0.016	<0.05
CENTER	ISLANDS	92	40	1546.5	2006.5	0.309	0.879	ns
SOUTH	ISLANDS	78	40	3482.5	2006.5	0.13	0.52	ns

**Table 7:** Descriptive statistics of the number of likes.

Variable	*n*	% Missing	Mean	SD	SE	Median	Range
Like number	379	0	8468.17	24,547.86	1260.94	1915.00	(1–193,510)

**Table 8:** Pairwise Wilcoxon rank-sum tests on the number of likes for the area.

Group 1	Group 2	n1	n2	Median 1	Median 2	*p*-Value	*p*-Value Adjusted	Significance
NORTH	CENTER	169	92	1768	1495	0.328	1	ns
NORTH	SOUTH	169	78	1768	3416	0.035	0.213	ns
NORTH	ISLANDS	169	40	1768	1972	0.73	1	ns
CENTER	SOUTH	92	78	1495	3416	0.003	0.015	<0.05
CENTER	ISLANDS	92	40	1495	1972	0.289	1	ns
SOUTH	ISLANDS	78	40	3416	1972	0.125	0.75	ns

## Data Availability

The data collected was acquired by consulting the official Facebook pages of Italian hospitals.

## Supplementary Materials

The following are available online at https://www.mdpi.com/article/10.3390/ijerph18147225/s1.
